# Microglia Regulate Pruning of Specialized Synapses in the Auditory Brainstem

**DOI:** 10.3389/fncir.2019.00055

**Published:** 2019-08-28

**Authors:** Giedre Milinkeviciute, Caden M. Henningfield, Michael A. Muniak, Sima M. Chokr, Kim N. Green, Karina S. Cramer

**Affiliations:** ^1^Department of Neurobiology and Behavior, University of California, Irvine, Irvine, CA, United States; ^2^Vollum Institute, Oregon Health & Science University, Portland, OR, United States; ^3^Hearing Research, Garvan Institute of Medical Research, Sydney, NSW, Australia; ^4^St Vincent’s Clinical School, UNSW Sydney, Sydney, NSW, Australia

**Keywords:** microglia, calyx of Held, MNTB, pruning, auditory brainstem, CSF1R inhibitors, depletion

## Abstract

The assembly of uniquely organized sound localization circuits in the brainstem requires precise developmental mechanisms. Glial cells have been shown to shape synaptic connections in the retinogeniculate system during development, but their contributions to specialized auditory synapses have not been identified. Here we investigated the role of microglia in auditory brainstem circuit assembly, focusing on the formation and pruning of the calyx of Held in the medial nucleus of the trapezoid body (MNTB). Microglia were pharmacologically depleted in mice early in development using subcutaneous injections of an inhibitor of colony stimulating factor 1 receptor, which is essential for microglia survival. Brainstems were examined prior to and just after hearing onset, at postnatal days (P) 8 and P13, respectively. We found that at P13 there were significantly more polyinnervated MNTB neurons when microglia were depleted, consistent with a defect in pruning. Expression of glial fibrillary acidic protein (GFAP), a mature astrocyte marker that normally appears in the MNTB late in development, was significantly decreased in microglia-depleted mice at P13, suggesting a delay in astrocyte maturation. Our results demonstrate that monoinnervation of MNTB neurons by the calyx of Held is significantly disrupted or delayed in the absence of microglia. This finding may reflect a direct role for microglia in synaptic pruning. A secondary role for microglia may be in the maturation of astrocytes in MNTB. These findings highlight the significant function of glia in pruning during calyx of Held development.

## Introduction

Binaural cues used for sound localization are initially processed in auditory brainstem circuits that require speed and reliability (Trussell, 1999). Accordingly, these pathways display specialized synapses. A key component is the calyx of Held (1893), the large, encapsulating excitatory axon terminal of globular bushy cells (GBC) in the anteroventral cochlear nucleus (AVCN) that terminates in the contralateral medial nucleus of the trapezoid body (MNTB; Morest, 1968; Smith et al., 1991; Kandler and Friauf, 1993). This synapse possesses several hundred active sites and a large readily releasable pool of synaptic vesicles that allow for fast, sustained, and precise signaling to MNTB neurons (Borst and Sakmann, 1996; Taschenberger et al., 2002).

**FIGURE 1:**
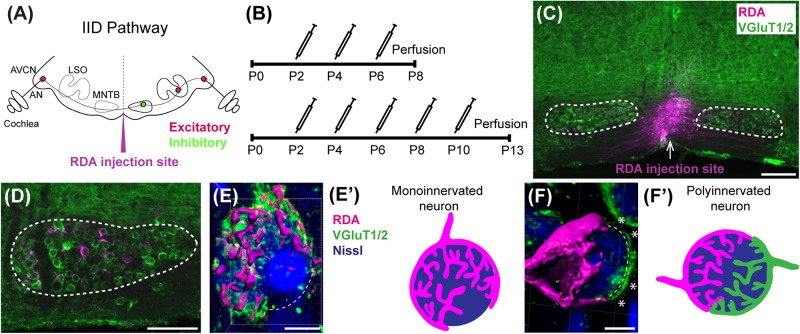
Calyx labeling, reconstruction in 3D, and analysis. **(A)** A schematic of the interaural intensity differences (IID) pathway and RDA injection site in the VAS to sparsely label calyces of Held in the MNTB. GBCs in the AVCN receive direct input from the AN. In turn, GBCs synapse on inhibitory neurons in the contralateral MNTB. MNTB neurons then innervate excitatory neurons in the LSO. LSO neurons also receive tonotopically matched excitatory terminals from SBCs in the ipsilateral AVCN. LSO neurons compute the balance of frequency-matched inhibition and excitation arising from both ears to determine the sound source location in the horizontal plane. **(B)** Timeline of injections. Two age cohorts were included in the study. The P8 mice group received vehicle or BLZ945 injections at P2, P4, and P6, and then were perfused at P8. Animals in the P13 age group were injected every 2 days from P2 until P10, and then perfused at P13. **(C)** A photograph of an RDA injection site (magenta) in the VAS and sparsely labeled calyces in the mouse brainstem. VGluT1/2 labeling is shown in green. **(D)** A higher magnification of the left MNTB with sparsely RDA-labeled calyces of Held. **(E)** Reconstructed calyx area used for size and volume measurements (magenta). MNTB neurons were classified into mono- or polyinnervated based on the presence of PV or VGluT1/2 labeling outside the RDA-labeled calyx. Shown is an example of a monoinnervated neuron. VGluT1/2 labeling is present and is co-localized to the RDA-labeled calyx. Dashed line indicates approximate boundary of the Nissl-stained neuron that is contacted by the RDA-filled calyx of Held. **(E’)** Schematic illustration of a monoinnervated MNTB neuron contacted by an RDA-labeled calyx. **(F)** An example of a polyinnervated MNTB neuron. It is contacted by an RDA-filled calyx and an additional VGluT1/2 positive calyceal input (indicated by white asterisks) around the remaining surface of the same neuron (dashed line). **(F’)** Schematic illustration of a polyinnervated MNTB neuron. Both the RDA-labeled calyx and one or more additional inputs terminate on the same MNTB neuron. Scale bar in panel **(C)** = 200 μm; scale bar in panel **(D)** = 100 μm; scale bar in panel **(E)** = 6 μm; scale bar in panel **(F)** = 5 μm. Abbreviations: AN – auditory nerve, AVCN – anterior ventral cochlear nucleus, GBC – globular bushy cell, LSO – lateral superior olive, MNTB – medial nucleus of the trapezoid body, SBC – spherical bushy cell, VAS – ventral acoustic stria. Panel **(A)** has been adapted from Joris and Yin (1998).

Medial nucleus of the trapezoid body neurons also receive other excitatory inputs such as collaterals of spherical bushy cells (Cant and Casseday, 1986), GBCs (Hamann et al., 2003), or descending input from higher auditory structures (Kuwabara et al., 1991) as well as inhibitory inputs (Albrecht et al., 2014). The MNTB is a sign-inverting relay nucleus that sends glycinergic inhibitory projections to several auditory brainstem nuclei, including the lateral superior olive (LSO; Kuwabara and Zook, 1991; Tollin, 2003) and medial superior olive (MSO; Spangler et al., 1985; Kuwabara and Zook, 1992) on the same side. The LSO also receives frequency-matched excitatory input from the ipsilateral AVCN (Sanes, 1990; Glendenning et al., 1992) and uses the balance of inhibitory MNTB and excitatory VCN inputs to compute interaural intensity differences used in sound localization (Figure 1A; Tollin, 2003; Kandler and Gillespie, 2005; Grothe et al., 2010). In the MSO, inhibitory input is needed for computation of interaural time differences, which provide sound localization cues for lower frequencies (Brand et al., 2002; Pecka et al., 2008; Myoga et al., 2014). The MNTB is thus a key relay nucleus for many aspects of auditory processing.

The calyx of Held undergoes major changes during development. In early postnatal development, axonal collaterals form multiple small “protocalyces” on MNTB neurons (Kandler and Friauf, 1993; Hoffpauir et al., 2006). These synapses are then refined and excess synapses are pruned until a single calyx remains on each MNTB neuron (Morest, 1968; Satzler et al., 2002; Hoffpauir et al., 2006; Holcomb et al., 2013). This pruning results in a largely one-to-one innervation pattern from the AVCN to the MNTB (Harrison and Warr, 1962; Friauf and Ostwald, 1988; Bergsman et al., 2004; Wimmer et al., 2004).

Communication between neurons and glia is required for the establishment of functional neural circuits (Reemst et al., 2016; Neniskyte and Gross, 2017). Microglia, the resident immune cells of the brain, are critical for brain wiring (Hammond et al., 2018) and synaptic pruning during development (Paolicelli et al., 2011; Ji et al., 2013). Their gene expression profiles, and likely their functions, vary with brain region (Grabert et al., 2016; De Biase and Bonci, 2018; Masuda et al., 2019). Microglia are most diverse in the developing mouse brain, with more homogenous populations present in the adult brain (Hammond et al., 2019; Li et al., 2019). We tested whether brainstem microglia contribute to the assembly of specialized auditory pathways. In the brainstem, microglia appear in the AVCN by postnatal day 0 (P0) and by P6 they are seen in the MNTB. Microglia peak in number during the second postnatal week, coinciding with the maturation of the calyx of Held. Several studies have shown that microglia influence expression of astrocytic proteins (Elmore et al., 2014; Spangenberg et al., 2016; Jin et al., 2017). Both astrocytes and microglia are found in close proximity to the developing calyx, indicating their possible involvement in its growth and maturation (Holcomb et al., 2013; Dinh et al., 2014).

Here, we treated animals during postnatal development with an inhibitor of colony stimulating factor 1 receptor (CSF1R), a tyrosine kinase receptor essential for microglia survival (Stanley et al., 1997; Erblich et al., 2011; Elmore et al., 2014). We demonstrated that this treatment effectively eliminated microglia during the postnatal period. We found that microglia depletion was associated with reduced expression of the mature astrocytic marker, glial fibrillary acidic protein (GFAP), suggesting a delay in astrocyte maturation. Significantly, elimination of microglia prevented the pruning of polyinnervated MNTB neurons. These results suggest that microglia are needed for synaptic pruning in the MNTB.

## Materials and Methods

### Animals

We used 42 postnatal day 8 (P8) and 46 P13 wild-type C57BL/6 mice of both sexes. All procedures were approved by the University of California, Irvine Institutional Animal Care and Use Committee.

### Treatment

Animals were treated with BLZ945 (MW: 398.48, MedChem Express HY-12768/CS-3971), a small molecule inhibitor of CSF1R (Pyonteck et al., 2013), to eliminate microglia. BLZ945 was dissolved in dimethylsulfoxide (DMSO; D136-1; Thermo Fisher Scientific) by gradually adding it to the vial with BLZ945 and briefly vortexing. 0.01 mL of solution was administered subcutaneously (sc) at a dose of approximately 200 mg/kg at P2. The same volume of drug was administered at P4 and P6 for the P8 endpoint. Additional injections were delivered at P8 and P10 for the animals perfused at P13. DMSO-only injections were used as controls (Figure 1B). At P8 or P13 mice were weighed and perfused transcardially with 0.9% saline followed by 4% paraformaldehyde (PFA) in 0.1 M phosphate buffer, pH 7.3 (PBS). Brainstems were dissected and postfixed in PFA solution. After 2–3 h, brains were equilibrated in a 30% sucrose solution in 0.1 M PBS then sectioned coronally at 18 μm using a cryostat (CM 1850-3-1; Leica Microsystems). Sections were mounted on chrome-alum coated glass slides in a 1-in-5 series.

### Neuronal Tracing

In 11 animals at P8 and 13 animals at P13 we performed neuronal tracing to fill calyces of Held in the MNTB. Mice were perfused transcardially with artificial cerebrospinal fluid (aCSF; 130 mM NaCl, 3 mM KCl, 1.2 mM KH_2_PO_4_, 20mM NaHCO_3_, 3 mM HEPES, 10 mM glucose, 2 mM CaCl_2_, 1.3 mM MgSO_4_ perfused with 95% O_2_ and 5% CO_2_). Brains were quickly dissected and placed in a chamber with oxygenated aCSF.

The brain was temporarily transferred to a Petri dish with aCSF and axonal projections from the AVCN to the MNTB were filled using a rhodamine dextran amine (RDA; MW 3000, Invitrogen) solution (6.35% RDA with 0.4% Triton-X100 in PBS). A pulled glass micropipette was filled with RDA and pulses of RDA were delivered close to the midline in the ventral acoustic stria (VAS) using an Electro Square Porator (ECM830; BTX) at a rate of 5 pulses per second (pps) at 55 V for 50 ms. These pulses resulted in sparse labeling of GBC axons and calyces of Held on both sides of the brainstem (Figures 1C,D). In some cases, sequential labeling was performed with Alexa 488 dextran amine (DA488; 6.35% with 0.4% Triton-X100 in PBS) so that a single brainstem would have AVCN axons sparsely labeled with two different fluorophores. The brain was then placed back into the aCSF chamber for approximately 2 h under continuous oxygenation to allow for dye transport. The tissue was then transferred to 4% PFA solution for another 2 h followed by incubation in 30% sucrose solution in 0.1 M PBS. Brainstems were cryosectioned in the coronal plane at 18 μm and mounted on an alternating series of four slides. Slides with adequately labeled axons and calyces of Held were immunolabeled for parvalbumin (PV) at P8 and for vesicular glutamate transporters 1 and 2 (VGluT1/2) at P13.

### Immunofluorescence

We performed immunofluorescence for a calcium binding protein specific for microglia (IBA1), VGluT1/2, vesicular GABA transporter (VGAT), synaptophysin1 (Syn), PV, and astrocytic markers – S100 calcium-binding protein B (S100β), aldehyde dehydrogenase 1 family member L1 (ALDH1L1), and GFAP.

Mounted sections were surrounded with a PAP pen hydrophobic barrier and rinsed in 0.1 M PBS for 10 min. For antigen retrieval, tissue was incubated for 5 min in 0.1% sodium dodecyl sulfate in 0.1 M PBS solution followed by washes in 0.1 M PBS. Sections were then blocked with normal goat blocking solution containing 5% normal goat serum (NGS; Vector Laboratories S-1000) and 0.3% Triton X-100 (Acros 9002-93-1) in 0.1 M PBS for 1 h in a humidity chamber at room temperature followed by overnight incubation in primary antibodies. The tissue was then rinsed in 0.1 M PBS and incubated for 1 h in goat anti-rabbit, anti-guinea pig, or anti-chicken secondary antibody tagged with an Alexa (Invitrogen) fluorophore. Sections were washed in 0.1 M PBS and incubated in red or blue fluorescent Nissl stain (NeuroTrace 530/615 or 435/455, Life Technologies N21482 or N21479) diluted 1:200 in 0.3% Triton X-100 in 0.1 M PBS. After 1 h, tissue was rinsed in 0.1 M PBS and coverslipped with Glycergel mounting medium (Dako C0563).

For PV labeling, tissue was incubated for 3 h in a blocking solution made of 10% bovine serum albumin (Sigma 1002552698), 5% NGS, and 0.1% Triton X-100 in 0.1 M PBS followed by a 48-h incubation with mouse anti-PV primary antibody at 4°C. Sections were then allowed to equilibrate at room temperature for 30 min and rinsed 5 times in 0.1 M PBS. Tissue was placed in the blocking solution for 30 min then incubated in goat anti-mouse secondary antibody tagged with an Alexa 488 fluorophore. Three hours later, slides were rinsed and incubated for 1 h in fluorescent Nissl stain diluted 1:200 in 0.3% Triton X-100 in 0.1 M PBS, washed 3 times and coverslipped with Glycergel mounting medium. The list of primary and secondary antibodies used in this study and their concentrations are listed in Table 1.

**TABLE 1:** List of antibodies used in the project.

**Antigen**	**Host**	**RRID**	**Cat. no.**	**Source**	**Dilution**
**Primary antibodies**					
ALDH1L1	Rabbit	N/A	ab177463	Abcam	1:500
IBA1	Rabbit	AB_839504	019-19741	Wako	1:500
GFAP	Chicken	AB_304558	ab4674	Abcam	1:1000
GlyT2	Rabbit	AB_2619997	272 003	Synaptic Systems	1:200
S100β	Rabbit	AB_882426	ab52642	Abcam	1:500
Synaptophysin	Guinea Pig	AB_1210382	101 004	Synaptic Systems	1:500
VGAT	Rabbit	AB_2492282	2100-VGAT	PhosphoSolutions	1:200
VGluT1/2	Rabbit	AB_2285905	135 503	Synaptic Systems	1:200
**Secondary antibodies**					
Alexa 488	Chicken	AB_2534096	A11039	Thermo Fisher Scientific	1:200
Alexa 488	Guinea Pig	AB_2534117	A11073	Thermo Fisher Scientific	1:500
Alexa 488	Rabbit	AB_2633280	A32731	Thermo Fisher Scientific	1:500
Alexa 647	Rabbit	AB_2535812	A21244	Thermo Fisher Scientific	1:500
Alexa 647	Guinea Pig	AB_2735091	A21450	Thermo Fisher Scientific	1:500

### Fluorescent Microscopy

We acquired 10× and 20× magnification images of sections throughout the rostro-caudal extent of the MNTB using a Zeiss Axioskop-2 microscope, an Axiocam camera, and Axiovision software. We analyzed the MNTB on both sides of the midline, including only sections with an intact MNTB. For each animal in our analysis, at least three sections were included per primary antibody.

### Microglial, Astrocytic, and Synaptic Protein Areal Coverage Analysis

For each specimen, a series of 20× multichannel fluorescent photographs spanning the rostro-caudal extent of the MNTB was assembled as an image stack in FIJI (Schindelin et al., 2012). For each section, the channel corresponding to Nissl-staining was used to outline the boundary of the MNTB, which was stored as a region-of-interest (ROI) polygon in FIJI. All subsequent operations were performed on the image channel corresponding to the immunolabeling of interest (e.g., VGAT). To correct for minor irregularities in overall illumination and/or labeling intensity across a set of sections, a level adjustment was applied such that the mean pixel intensity within each MNTB ROI was equivalent across all sections of an image stack without causing over- or undersaturation. Only global (section-wide) adjustments of pixel values were permitted; local manipulations were not made.

To identify positive immunolabeling, a histogram of pixel intensity was constructed based on all pixels within all MNTB ROIs in the image stack. This histogram was used as the input for automatic image thresholding in FIJI where a threshold value was computed using the *Default* algorithm and applied to all images in the stack. Label/optical density for each section was evaluated and the areal coverage ratio was quantified as the total number of thresholded pixels within a given MNTB ROI.

### Microglia Counts

Each section used for IBA1 areal coverage analysis was examined for microglial cell counts. Microglial cell bodies were identified by a small cell body with processes radiating out of it. Microglia were not counted if only IBA1-positive processes but no somata were present. If a microglial cell body was on the edge of the MNTB ROI (drawn with reference to Nissl staining), it was excluded if <50% of the soma was inside of the ROI. IBA1-positive cells were counted using FIJI’s “Cell Counter” plugin. The average number of cells per MNTB slice was calculated by dividing the microglia count by the number of MNTB slices examined.

### Confocal Microscopy

Rhodamine dextran amine-labeled and PV- (P8) or VGluT1/2- immunolabeled (P13) tissue was used for confocal microscopy (Leica SP8, 63× oil objective, zoom: 1.5, pinhole: 1) and subsequent analysis. Nissl, RDA, and PV or VGluT1/2 z-stack images of calyces of Held were acquired at a resolution of 1024 × 1024 pixels, with a z-step size of 0.5 μm (Grande et al., 2014; Wang et al., 2018). Gain and offset were set for each fluorescent channel and each slide separately. In a few cases settings were adjusted to account for differences in labeling intensity between sections on the same slide. Image stacks were exported and the surface area and volume of calyces of Held were analyzed using the surface module in Imaris software (v9.2.1; Bitplane). Calyces throughout the MNTB were randomly labeled with RDA and, thus, the entire medio-lateral extent of the MNTB was analyzed. Surfaces of RDA-labeled calyces of Held were reconstructed using the default mode and then manually adjusted to achieve as accurate RDA fill of the calyx of Held as possible (Figure 1). Sections were coded and analyzed blind to treatment group. We did not change the intensity of confocal stacks to avoid affecting surface area and volumetric results. Only complete or near complete RDA-filled calyces of Held with visible labeled preterminal axon segments were included in the analysis. However, the preterminal axon itself was cropped out of the images where possible. Additionally, RDA-labeled calyces that were too close to one another to be individually reconstructed were excluded from the analysis. All calyces that matched the criteria were analyzed from at least one slide for each animal used. If the number of labeled calyces from one slide was too small (<3), an additional slide was used, however, care was taken to avoid examining serial sections (Table 2).

**TABLE 2:** Numbers of calyces of Held analyzed.

**Treatment**	**Age**	**Animals used**	**Animal ID**	**Calyces measured**	**N neurons scored for innervation**	**N polyinnervated neurons**
DMSO	P8	6	B143	12	15	13
			B144	8	13	11
			B153	13	11	11
			B156	9	15	10
			B160	11	15	15
			B216	8	9	6
BLZ945	P8	5	B141	13	10	9
			B149	10	22	16
			B154	11	14	11
			B217	12	7	4
			B215	19	23	14
DMSO	P13	6	B432	3	4	1
			B438	10	11	1
			B455	10	12	1
			B459	7	6	0
			B460	8	10	0
			B461	8	10	2
BLZ945	P13	7	B429	5	6	5
			B437	9	11	5
			B439	6	10	5
			B454	9	12	2
			B456	11	12	5
			B457	11	15	4
			B458	11	9	3

### Determination of Monoinnervation

At the time of surface rendering, the mono- or polyinnervation status of the postsynaptic neuron was evaluated. Sparse RDA-labeled brainstems were immunolabeled for PV (P8) or VGluT1/2 (P13) and MNTB cells with RDA-labeled input were analyzed. For a monoinnervated MNTB neuron, there would be no PV or VGluT1/2 labeling outside an RDA-labeled calyx (Figures 1E,E’). If additional inputs were present, we would see PV or VGluT1/2 along the surface of the MNTB neuron but outside the RDA-labeled calyx (Figures 1F,F’). MNTB neurons normally transition through a period in which a single dominant input is seen as early as P6 (Holcomb et al., 2013) and thus smaller calyces may be transiently present. However, small areas of spurious labeling with presynaptic markers outside the RDA label would not necessarily correspond to additional calyces (Cant and Casseday, 1986; Kuwabara et al., 1991; Smith et al., 1998). We set a threshold such that if PV or VGluT1/2 labeling outside the RDA-labeled calyx covered 25% or more of the MNTB cell surface, we considered that MNTB cell to be polyinnervated. This percent coverage was an estimated subjective score made blind to treatment group, and was roughly the size of the smallest calyces we labeled (see section “Results”). We then determined the percentage of analyzed MNTB neurons that were polyinnervated for each group (Table 2).

### Neuronal Density

To test for effects of drug treatment on cell number, we determined the cell density in AVCN (*n* = 4 DMSO; *n* = 4 BLZ945) and MNTB (*n* = 9 DMSO; *n* = 9 BLZ945) at P13. Tissue was prepared as described previously and cells were plotted using blue fluorescent Nissl staining. Fiji’s “Cell Counter” was used to count neurons within the ROI of each AVCN and MNTB (see above). Principle neurons of the MNTB were identified as homogenous cells that were round or slightly oval in shape and similar in size (Hoffpauir et al., 2006; Weatherstone et al., 2017). Only neuronal bodies with a visible nucleus and nucleolus were counted in every fifth section throughout the MNTB on both sides of the midline, including only intact MNTB sections. Neuronal density was calculated by dividing the sum of neuronal counts from each MNTB by the sum of MNTB area in the slices used for analysis.

### VGluT1/2 Co-localization With EGFP-Labeled Microglia

We performed VGluT1/2 immunofluorescence in *CX_3_CR1*^+/EGFP^ reporter mice to investigate a possible internalization of presynaptic proteins in microglia. These mice have enhanced green fluorescent protein (EGFP) inserted into exon 2 of *CX_3_CR1*, resulting in EGFP-positive microglial cells (Jung et al., 2000). We used 3 heterozygous mice at P8 and 3 at P13. Animals were perfused as described above and the same immunolabeling protocol was used to label for VGluT1/2. High resolution confocal imaging was used to build z-stacks through the microglial cell (Leica SP8, 63× oil objective, zoom: 3.5, pinhole: 0.6, resolution of 2048 × 2048 pixels, z-step size of 0.5 μm). Subsequently, z-stacks were deconvolved using Huyens essentials software and examined using Imaris. Due to the nature of on-slide immunolabeling, VGluT1/2 antibody penetration was not always complete, thus, z-stacks were cropped so that EGFP-positive microglia were in the same focal plane as VGluT1/2 labeling. A co-localization function in Imaris was used to build the co-localization channel and thresholds of overlap of EGFP and VGluT1/2 channels were set to 10% and kept the same for all microglial cells analyzed. A total of five microglial cells per animal were imaged from non-consecutive sections, thus, 15 cells per age group in total.

### Software Accessibility

FIJI scripts will be made available upon request to Dr. Michael A. Muniak.

### Experimental Design and Statistical Analysis

To account for variability among litters, multiple litters were used and each of them was divided into control and experimental animals. Mice for each cohort were allocated randomly. Quantitative results for all cell counts, calyx of Held sizes, neuronal density, and innervation status as well as coverage of positive immunohistochemistry are represented by mean scores ± standard error of mean (SEM) and were analyzed using Prism (v7; GraphPad Software). Comparisons between different treatment and age groups were made using a two-way ANOVA and Sidak’s multiple comparisons test unless stated otherwise. Statistical significance was accepted at *p* < 0.05. Details of statistical analysis are presented in Table 3.

**TABLE 3:** Statistical analysis.

**Figures**	**Sample size (*n*)**	**Mean ± SEM**	**Treatment effect** **(DMSO-BLZ945)**	**Age effect (P8-P13)**
Figure 2J	DMSO: P8 = 11, P13 = 11; BLZ945: P8 = 13, P13 = 9	DMSO: P8 0.01 ± 0.001, P13 0.04 ± 0.01; BLZ945: P8 0 ± 0, P13 0.001 ± 0.0002	P8: *p* = 0.0036, DF = 40; P13: *p* < 0.0001, DF = 40	DMSO: *p* < 0.0001, DF = 40; BLZ945: *p* = 0.9869, DF = 40

Figure 2K	DMSO: P8 = 11, P13 = 10; BLZ945: P8 = 13; P13 = 9	DMSO: P8 3.41 ± 0.33, P13: 5.78 ± 0.30; BLZ945: P8 0 ± 0, P13 0.25 ± 0.06	P8: *p* < 0.0001, DF = 39; P13: *p* < 0.0001, DF = 39	DMSO: *p* < 0.0001, DF = 39; BLZ945: *p* = 0.6644, DF = 39

Figure 2L	DMSO: P8 = 11, P13 = 10; BLZ945: P8 = 14; P13 = 9	DMSO: P8 4.28 ± 0.14, P13: 7.22 ± 0.19; BLZ945: P8 3.67 ± 0.21, P13 5.36 ± 0.26	P8: *p* = 0.0574, DF = 40; P13: *p* < 0.0001, DF = 40	DMSO: *p* < 0.0001, DF = 40; BLZ945: *p* < 0.0001, DF = 40

Figure 2M	DMSO: P8 = 11, P13 = 10; BLZ945: P8 = 14; P13 = 9	DMSO: P8 0.31 ± 0.01, P13: 0.41 ± 0.01; BLZ945: P8 0.30 ± 0.01, P13 0.40 ± 0.01	P8: *p* = 0.2951, DF = 39; P13: *p* = 0.5375, DF = 39	DMSO: *p* < 0.0001, DF = 39; BLZ945: *p* < 0.0001, DF = 39

Figure 3M	DMSO: P8 = 8, P13 = 10; BLZ945: P8 = 10; P13 = 8	DMSO: P8 0.24 ± 0.02, P13: 0.26 ± 0.01; BLZ945: P8 0.26 ± 0.01, P13 0.26 ± 0.02	P8: *p* = 0.2971, DF = 38; P13: *p* = 0.9982, DF = 38	DMSO: *p* = 0.5207, DF = 38; BLZ945: *p* = 0.8819, DF = 38

Figure 3N	DMSO: P8 = 11, P13 = 10; BLZ945: P8 = 12; P13 = 9	DMSO: P8 0.23 ± 0.01, P13: 0.27 ± 0.01; BLZ945: P8 0.24 ± 0.01, P13 0.27 ± 0.01	P8: *p* = 0.8814, DF = 32; P13: *p* > 0.9999, DF = 32	DMSO: *p* = 0.0667, DF = 32; BLZ945: *p* = 0.1707, DF = 32

Figure 3O	DMSO: P8 = 11, P13 = 10; BLZ945: P8 = 12; P13 = 9	DMSO: P8 0.02 ± 0, P13: 0.04 ± 0; BLZ945: P8 0.02 ± 0, P13 0.02 ± 0	P8: *p* = 0.9307, DF = 38; P13: *p* = 0.0042, DF = 38	DMSO: *p* < 0.0001, DF = 38; BLZ945: *p* = 0.5687, DF = 38

Figure 5M	DMSO: P8 = 11, P13 = 10; BLZ945: P8 = 13; P13 = 9	DMSO: P8 0.18 ± 0.01, P13: 0.17 ± 0.01; BLZ945: P8 0.19 ± 0.01, P13 0.19 ± 0.01	P8: *p* = 0.3952, DF = 39; P13: *p* = 0.3401, DF = 39	DMSO: *p* = 0.7678, DF = 39; BLZ945: *p* = 0.9012, DF = 39

Figure 5N	DMSO: P8 = 11, P13 = 7; BLZ945: P8 = 12; P13 = 9	DMSO: P8 0.16 ± 0.005, P13: 0.22 ± 0.02; BLZ945: P8 0.15 ± 0.01, P13 0.20 ± 0.02	P8: *p* = 0.8088, DF = 35; P13: *p* = 0.5781; DF = 35	DMSO: *p* = 0.0011, DF = 35; BLZ945: *p* = 0.0018, DF = 35

Figure 5O	DMSO: P8 = 11, P13 = 10; BLZ945: P8 = 13; P13 = 9	DMSO: P8 0.23 ± 0.005, P13: 0.27 ± 0.01; BLZ945: P8 0.24 ± 0.01, P13 0.26 ± 0.02	P8: *p* = 0.8992, DF = 39; P13: *p* = 0.9016, DF = 39	DMSO: *p* = 0.0328, DF = 39; BLZ945: *p* = 0.1836, DF = 39

Figure 7A	DMSO: P8 = 6, P13 = 6; BLZ945: P8 = 5; P13 = 7	DMSO: P8 84.10% ± 6.11%, P13 10.40% ± 4.19%; BLZ945: P8 71.86% ± 5.97%, P13 42.45% ± 8.07%	P8: *p* = 0.3914, DF = 20; P13: *p* = 0.0035, DF = 20	DMSO: *p* < 0.0001, DF = 20; BLZ945: *p* = 0.0101, DF = 20

Figure 7B	AVCN DMO: P13 = 4; AVCN BLZ945: P13 = 4; MNTB DMSO: P13 = 9; MNTB BLZ945: P13 = 9	AVCN DMSO: P13 0.0015 ± 0.0001; AVCN BLZ945:P13 0.0016 ± 0.0001; MNTB DMSO: P13 0.0013 ± 0.00004932; MNTB BLZ945: P13 0.0014 ± 0.00003985	P13 AVCN: *p* = 0.8693, *t* = 0.1717; DF = 6; P13 MNTB: *p* = 0.1052, *t* = 1.717; DF = 16	N/A

Figure 7C	DMSO: P8 = 6, P13 = 6; BLZ945: P8 = 5; P13 = 7	DMSO: P8 654.5 μm^2^ ± 70.56 μm^2^, P13: 947.7 μm^2^ ± 110.6 μm^2^; BLZ945: P8 702.6 μm^2^ ± 58.57 μm^2^, P13 896.6 μm^2^ ± 32.52 μm^2^	P8: *p* = 0.8840, DF = 20; P13: *p* = 0.8480, DF = 20	DMSO: *p* = 0.0191, DF = 20; BLZ945: *p* = 0.1470, DF = 20

Figure 7D	DMSO: P8 = 6, P13 = 6; BLZ945: P8 = 5; P13 = 7	DMSO: P8 237 μm^3^ ± 28.64 μm^3^, P13: 358.9 μm^3^ ± 53.67 μm^3^; BLZ945: P8 242.6 μm^3^ ± 22.19 μm^3^, P13 318.5 μm^3^ ± 19.9 μm^3^	P8: *p* = 0.9924, DF = 20; P13: *p* = 0.6299, DF = 20	DMSO: *p* = 0.0380, DF = 20; BLZ945: *p* = 0.2487, DF = 20

## Results

### Microglia Depletion With BLZ945

**FIGURE 2:**
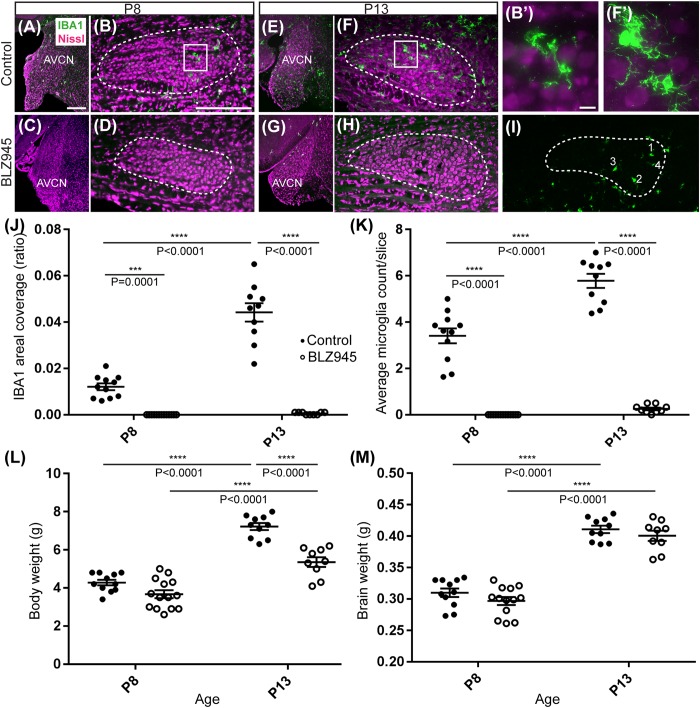
Microglia depletion with BLZ945. **(A)** Microglia (green) are observed within the AVCN and **(B)** MNTB (dashed line) at P8 in control mice. **(B’)** Inset from panel **(B)**. A higher magnification of the IBA1-labeled microglia from the MNTB of a control mouse at P8. **(C)** BLZ945 treatment eliminates microglia in the AVCN and **(D)** MNTB at P8. **(E)** At P13 microglia are more abundant than at P8 in control AVCN and **(F)** MNTB. **(F’)** Inset from panel **(F)**. A higher magnification of the IBA1-labeled microglia from the MNTB of a control mouse at P13. **(G)** BLZ945 treatment eliminates microglia almost completely in the AVCN and **(H)** MNTB at P13. **(I)** Microglia counts in MNTB. Example of how microglial cell bodies were identified within the MNTB ROI. **(J)** Areal coverage ratio of IBA1 immunolabel. Microglia are significantly reduced at P8 and P13 in BLZ945-injected mice when compared to their age-matched controls. Microglia significantly increase with age in control animals but they remain almost absent in the MNTB of BLZ945-injected mice. **(K)** Microglia numbers per MNTB slice are significantly larger in control than BLZ945-treated mice at P8 and P13. Microglia increase in number in control animals with age but this increase was not observed in the BLZ945 injected group. **(L)** Control and BLZ945 injected animals gain weight with age, but mice without microglia weigh significantly less than their control littermates at P13. **(M)** Brains of control and BLZ945 injected animals significantly increased in weight with age. There was no significant difference between the brain of DMSO- and BLZ945-injected mice at P8 and P13. Scale bar in panel **(A)** = 100 μm, applies to panels **(A,C,E,G)**. Scale bar in panel **(B)** = 200 μm, applies to panels **(B,D,F,H,I)**. Scale bar in panel **(B’)** = 10 μm and applies to the panel **(F’)**.

Two age groups of mice were selected for the experiment: P8 and P13, corresponding to ages before and after hearing onset, respectively. A total of 38 mice were used for the P8 age cohort, of which 20 were injected with DMSO and 18 with BLZ945. A total of 46 animals were included in the P13 age group, of which 26 were injected with DMSO and 20 with BLZ945. Littermate controls were used in each age cohort. BLZ945-treated mice were smaller and had some craniofacial changes, such as shorter snouts and smaller teeth, than their control littermates. This effect is akin to the phenotype seen in CSF1R KO mice (Dai et al., 2002). Occasionally, BLZ945 treatment resulted in loss of pigment, in particular, in areas around the ears and neck. Overall, survival rates were near 100% for DMSO and 75% for BLZ945. We examined expression patterns of microglial marker IBA1 in response to vehicle or BLZ945 injections. At P8 we found microglia distributed throughout the extent of the AVCN and MNTB in the control group (Figures 2A,B). However, three subcutaneous injections (at P2, P4, and P6) of BLZ945 in developing pups, in most cases, completely depleted microglia in the brainstems of the P8 mice (Figures 2C,D). At P13, microglia were more numerous in the MNTB of the vehicle treated mice (Figures 2E,F), while the experimental group showed very little if any IBA1-positive labeling (Figures 2G,H). Thus, BLZ945 injections successfully depleted microglia in the MNTB and its presynaptic nucleus, the AVCN. We did not observe any apparent cellular debris from microglia, likely because the BLZ945 treatment was initiated before microglia normally populate the MNTB (Dinh et al., 2014). Cellular debris was also not present in adult mice after a few days of treatment with CSF1R inhibitors (Elmore et al., 2014).

We quantified IBA1 labeling in the MNTB using the areal coverage ratio of immunolabeling, as described in section “Materials and Methods.” BLZ945 treatment resulted in a significant decrease in microglia coverage in both P8 and P13 pups when compared to their age-matched littermate controls (P8: *p* = 0.0036; P13: *p* < 0.0001). Microglial coverage significantly increased with age in the control group (*p* < 0.0001; Figure 2J). Consequently, repeated BLZ945 delivery during the first two postnatal weeks depleted microglia and prevented them from reappearing by 3 days after the last injection (at P10). The same IBA1-labeled tissue was used to calculate the average number of microglial cells per MNTB slice (Figures 2I,K). At P8, there were significantly more microglia present in control than in BLZ945-treated mice (*p* < 0.0001). The difference remained significant after hearing onset (*p* < 0.0001). Additionally, microglial count increased with age in the control group (*p* < 0.0001) while there was no change observed between the two BLZ945 injected groups (*p* = 0.6644).

Mice treated with BLZ945 appeared smaller than their age-matched littermates, thus, we compared the weights of animals used for the experiment and also measured their brain weight. While there was no significant difference observed at P8 in animal weight (*p* = 0.0574), microglia depleted animals sacrificed at P13 weighed significantly less than DMSO injected controls (*p* < 0.0001). However, both vehicle and CSF1R inhibitor injected mice were significantly heavier after the onset of hearing than before (*p* < 0.0001 and *p* < 0.0001, respectively; Figure 2L). Consequently, both experimental groups gained weight with age but animals without microglia gained significantly less. Interestingly, there was no significant difference between the brain weight of control and BLZ945-injected mice at P8 (*p* = 0.2951) and P13 (*p* = 0.5375). Brain weight of mice in both treatment groups significantly increased with age (DMSO: *p* < 0.0001; BLZ945: *p* < 0.0001; Figure 2M).

### BLZ945 Effect on Astrocytes

**FIGURE 3:**
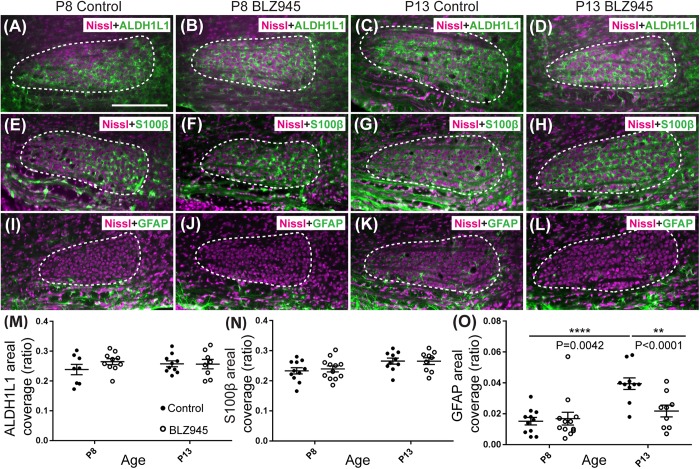
Effects of microglia depletion on different astrocytic markers. Images of the MNTB (dashed line) from **(A)** control and **(B)** BLZ945-treated mice at P8 showing Nissl staining (magenta) and ALDH1L1 (green) expression. **(C)** Images from control mice and **(D)** BLZ945-injected mice at P13 show ALDH1L1 expression similar to that seen at P8 throughout the MNTB. **(E)** Image of an MNTB section from a control mouse at P8 showing Nissl (magenta) and S100β labeling (green). Like ALDH1L1, S100β-positive astrocytes are abundant throughout the MNTB. **(F)** Image from a BLZ945-treated mouse at P8 appears similar to age-matched control in panel **(E)**. Images of the MNTB from **(G)** control and **(H)** BLZ945-injected mice at P13. **(I)** Image of the MNTB from a control mouse at P8 showing Nissl and GFAP labeling. GFAP-positive astrocytes at this age are sparse and limited to the boundaries of the MNTB. **(J)** Image of the MNTB from a BLZ945-treated mouse at P8 shows that, like controls, GFAP-positive astrocytes are sparse in the MNTB. **(K)** Image from a control mouse at P13. At this age GFAP is found throughout the MNTB. **(L)** Image of the MNTB section from a BLZ945-treated mouse at P13. GFAP immunolabel is less abundant than in control animals. There was no significant difference in areal coverage of both **(M)** ALDH1L1 and **(N)** S100β labeling in the MNTB between control and microglia-depleted animals within and between different age groups. **(O)** Areal coverage ratio of GFAP labeling in the MNTB. The GFAP percent coverage in the MNTB significantly increased with age in control animals. There was no significant change in GFAP levels in microglia-depleted mice with age. At P13, the areal GFAP labeling in the MNTB of BLZ945-treated mice was significantly less than that seen in control P13 animals. Scale bar in panel **(A)** = 200 μm, applies to panels **(A–L)**.

Three antibodies for astrocytic markers previously shown to be expressed in the mouse brainstem (Dinh et al., 2014) were used to label astrocytes in the MNTB before and after hearing onset. S100β and ALDH1L1 are early appearing markers in the MNTB, whereas GFAP appears later (Dinh et al., 2014). Tissue from DMSO- and BLZ945-injected mice was analyzed in the same way as that for IBA1 as described in section “Materials and Methods.” Numerous S100β and ALDH1L1 positive astrocytes were present throughout the MNTB of both control and experimental animals at P8 and P13. ALDH1L1 and S100β immunolabeling appeared to be very similar with both types of astrocytes surrounding neuronal somata (Figures 3A–H). After quantifying areal coverage of ALDH1L1 and S100β labeling in the MNTB, no significant difference was observed between the control and treated mice at P8 (ALDH1L1: *p* = 0.2971; S100β: *p* = 0.8814) or at P13 (ALDH1L1: *p* = 0.9982; S100β: *p* > 0.9999). Additionally, the amount of these proteins remained very similar before and after hearing onset (ALDH1L1: DMSO *p* = 0.5207; BLZ945 *p* = 0.8819; S100β: DMSO *p* = 0.0667; BLZ945 *p* = 0.1707; Figures 3M,N).

As previously shown (Dinh et al., 2014), immunolabeling for the mature astrocyte marker GFAP was minimal at P8 (Figures 3I,J). At P13, GFAP immunolabeling could be seen in the MNTB in control mice (Figure 3K) but relatively little was seen in the MNTB of experimental animals (Figure 3L). Quantification of GFAP areal coverage revealed no difference between the control and BLZ945-treated groups at P8 (*p* = 0.9307). GFAP-positive MNTB area significantly increased with age in control (*p* < 0.0001) but not experimental animals (*p* = 0.5687). At P13, we found significantly smaller GFAP areal coverage in the MNTB of microglia-depleted mice than in control mice (*p* = 0.0042; Figure 3O).

**FIGURE 4:**
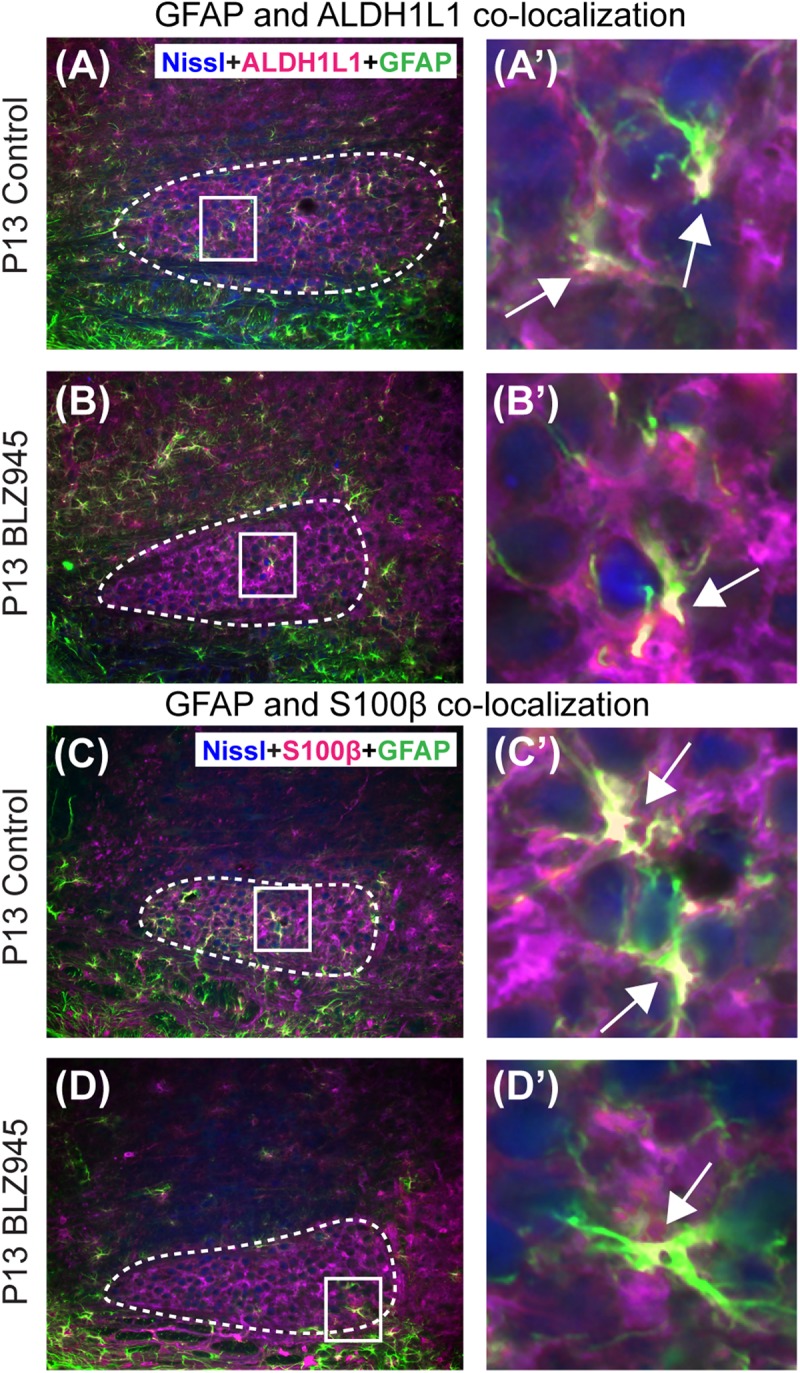
Co-localization of GFAP with different astrocytic markers. **(A)** GFAP is expressed throughout the MNTB (dashed line) in control animals at P13. **(A’)** Inset from panel **(A)**. White color and arrows indicate the areas of GFAP and ALDH1L1 co-localization. **(B)** In the absence of microglia, GFAP expression in the MNTB is sparse. **(B’)** Inset from panel **(B)**. Despite the scant presence of GFAP, co-localization between the two astrocytic markers is observed. **(C)** GFAP in control animals also co-localizes with S100β. **(C’)** Inset from panel **(C)** showing a higher magnification of co-localization. **(D)** Microglial depletion reduces the expression of GFAP but co-localization is still evident. **(D’)** Inset from panel **(D)** is a higher magnification of an area showing co-localized GFAP and S100β in the MNTB. Scale bar in panel **(A)** = 100 μm, applies to panels **(A–D)**. Scale bar in panel **(A’)** = 25 μm, applies to panels **(A’–D’)**.

Glial fibrillary acidic protein may be a marker for mature astrocytes (Gomes et al., 1999). We used 5 DMSO and 4 BLZ945-injected mice to label the tissue for GFAP and ALDH1L1 at P13. We examined MNTB area for co-localization between the two astrocytic markers (Figures 4A,B). A high degree of co-localization was observed in both control and microglia depleted animals. The same effect was seen when tissue from 10 DMSO and 9 BLZ945-treated mice was labeled for GFAP and S100β (Figures 4C,D). Taken together, the results do not show effects of microglia depletion on astrocytes during early development, but rather that removal of microglia may impede the maturation of astrocytes.

### BLZ945 Effect on Synaptic Proteins

**FIGURE 5:**
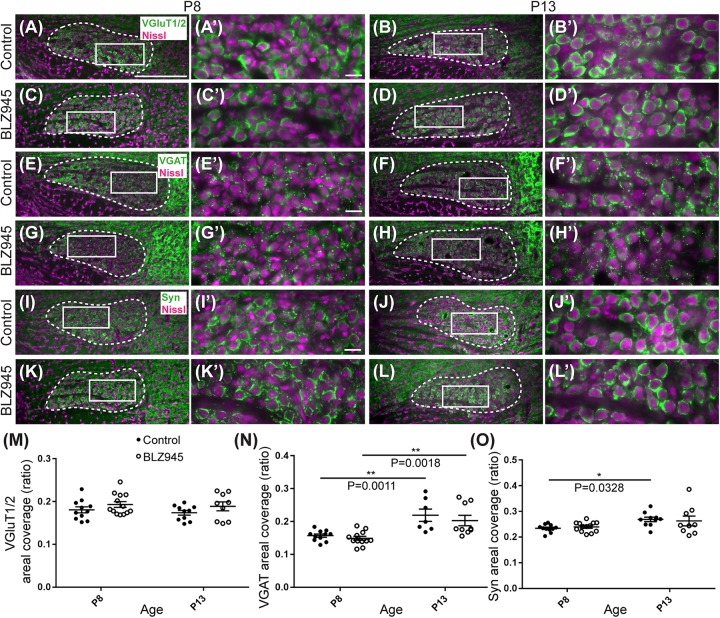
Effects of microglia depletion on synaptic protein expression in the MNTB. **(A)** Images of the MNTB (dashed line) from control mice at P8 and **(B)** at P13. Nissl-stained cell bodies are shown in magenta; VGluT1/2 immunolabel is shown in green. Panels **(A’,B’)** are higher magnifications of insets in panels **(A,B)**, respectively. **(C)** VGluT1/2 labeling in the MNTB from BLZ945-treated mice at P8 and **(D)** at P13. Panels **(C’,D’)** are higher magnifications of insets in panels **(C,D)**, respectively. VGluT1/2 labeling appears to be comparable throughout all panels. **(E)** Images of the MNTB (dashed line) from control mice at P8 and **(F)** at P13. Nissl-stained cell bodies are shown in magenta; VGAT labeling is shown in green. Panels **(E’,F’)** are higher magnifications of insets in panels **(E,F)**, respectively. **(G)** VGAT labeling in the MNTB from BLZ945-treated mice at P8 and **(H)** at P13. Panels **(G’,H’)** are higher magnifications of insets in panels **(G,H)**, respectively. VGAT immunolabel is similar throughout all panels. **(I)** Images of the MNTB (dashed line) from control mice at P8 and **(J)** at P13. Nissl-stained neurons are shown in magenta; Syn label is shown in green. Panels **(I’,J’)** are higher magnifications of insets in panels **(I,J)**, respectively. **(K)** Syn labeling in the MNTB from BLZ945-treated mice at P8 and **(L)** at P13. Panels **(K’,L’)** are higher magnifications of insets in panels **(K,L)**, respectively. Syn labeling is similar throughout all panels. **(M)** Areal coverage ratio of VGluT1/2 labeling in the MNTB. There was no significant difference between control and microglia-depleted animals before or after hearing onset as well as between different age groups. **(N)** Areal coverage ratio of VGAT in the MNTB. There was a significant increase in VGAT labeling with age in both control and microglia-depleted mice. **(O)** Areal coverage ratio of Syn labeling in the MNTB. There was a significant increase in Syn expression with age in control mice. No difference was observed between control and BLZ945-treated mice. Scale bar in panel **(A)** = 200 μm, applies to panels **(A–L)**. Scale bar in panel **(A’)** = 20 μm, applies to panels **(A’–L’)**.

The MNTB receives the majority of its excitatory input from the AVCN (Billups, 2005) and inhibitory input from the ventral nucleus of the trapezoid body (Albrecht et al., 2014). To evaluate the expression of synaptic inputs in the presence or absence of microglia, we carried out immunofluorescence labeling for VGluT1/2 and VGAT which correspond to excitatory (Fremeau et al., 2001; Herzog et al., 2001; Kaneko et al., 2002) and inhibitory (McIntire et al., 1997; Wang et al., 2009) neurotransmitter transporters, respectively. Additionally, we immunolabeled for Syn, a presynaptic protein associated with small synaptic vesicles (Ngsee et al., 1990; Calakos and Scheller, 1994) to represent combined presynaptic inputs (Figure 5).

VGluT1/2 labeling in the MNTB of mature animals produces ring-like structures as calyces of Held nearly completely surround MNTB neurons. We quantified the coverage of VGluT1/2 and found no significant difference when compared to their littermate controls at P8 (*p* = 0.3952) or P13 (*p* = 0.3401). Comparisons between the two age groups did not show any significant change in VGluT1/2 expression either in control (*p* = 0.7678) or in treated animal groups (*p* = 0.9012; Figure 5M). Thus, microglia depletion did not significantly impact the expression of VGluT1/2.

While excitatory input dominates MNTB neurons (Billups, 2005), VGAT expression was observed in the MNTB at P8 and P13. Inhibitory inputs contacted mainly neuronal somata (Figures 5E–H’). VGAT coverage in BLZ945-treated mice did not differ significantly from control mice at P8 (*p* = 0.8088) or at P13 (*p* = 0.5781). However, in both control and treated animal groups, VGAT coverage significantly increased with age (*p* = 0.0011 and *p* = 0.0018, respectively; Figure 5N). Similarly, Syn levels showed no significant difference between control and BLZ945 groups at both P8 (*p* = 0.8992) and P13 (*p* = 0.9016). On the other hand, we found a significant increase in Syn levels in control mice between P8 and P13 (*p* = 0.0328). No age effect was observed in BLZ945-treated animals (*p* = 0.1836; Figure 5L). Taken together, these observations suggest that microglia are not needed for normal levels of synaptic protein expression before or after hearing onset.

### Microglia Depletion Impairs Pruning of Excess Calyces

**FIGURE 6:**
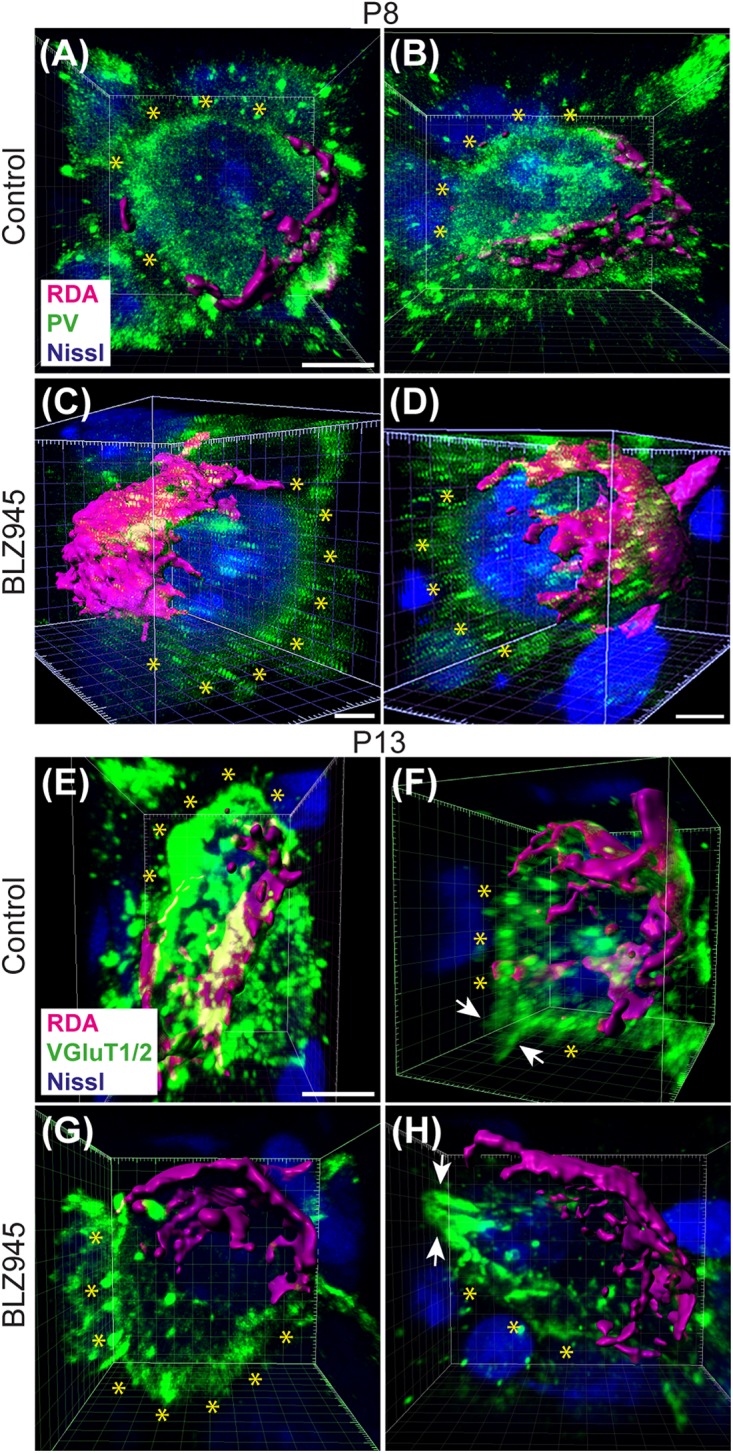
Confocal analysis of polyinnervated neurons in the presence or absence of microglia. **(A,B)** Images showing reconstructed RDA-labeled calyces of Held (magenta) terminating on MNTB principal neurons in control mice at P8. Both are examples of polyinnervated MNTB neurons as evidenced by RDA-labeled calyces and additional PV (green) labeling (asterisks) outside of RDA-labeled calyces. **(C,D)** Examples of polyinnervated neurons in BLZ945-treated mice at P8. **(E,F)** Examples of polyinnervated neurons in control mice at P13. Asterisks indicate VGluT1/2 labeling on neurons in addition to RDA-filled calyces of Held. **(G,H)** Images of polyinnervated neurons in BLZ945-treated mice at P13. Scale bar in panel **(A)** = 3 μm, applies to panels **(A,B)**. Scale bar in panels **(C,D)** = 3 μm. Scale bar in panel **(E)** = 3 μm, applies to panels **(E–H)**.

**FIGURE 7:**
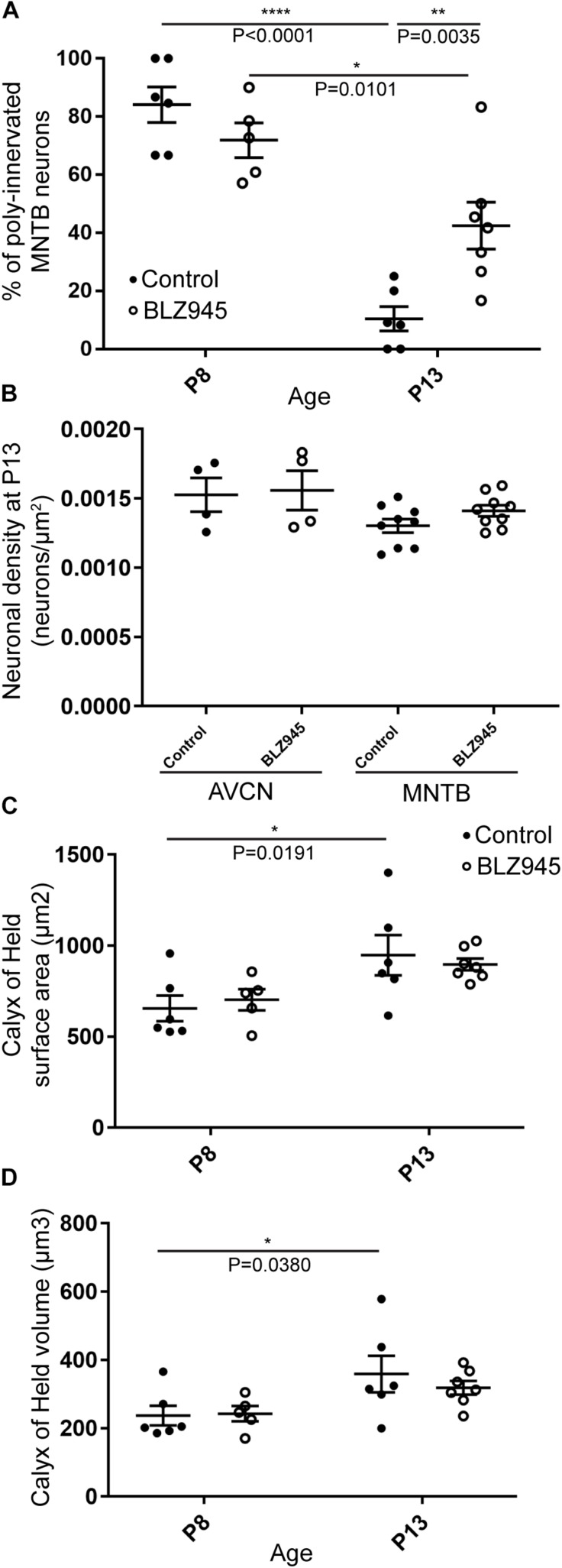
BLZ945 effect on the innervation of MNTB neurons and the size of the calyx of Held. **(A)** Percentage of polyinnervated MNTB neurons was calculated for each group. Before hearing onset, most of neurons in the MNTB were polyinnervated in both control and BLZ945-injected animals. The number of polyinnervated neurons significantly decreased at P13 in both experimental groups, but there were significantly more polyinnervated neurons remaining in microglia-depleted mice. **(B)** Neuronal density in AVCN and MNTB was calculated in control and microglia-depleted animals at P13 and there was no significant difference found between the two cohorts in either of the nuclei analyzed. **(C)** The surface area of calyces of Held was measured after 3D reconstruction of confocal z-stacks. There was no difference in surface area between control and BLZ945-treated mice at P8 or P13. In control animals, calyces grew significantly larger with age. **(D)** The volume of calyces of Held was also measured and was similar in control and treated animals at both ages. The volume of calyces significantly increased with age only in the control group.

We evaluated the innervation status of MNTB neurons in the absence of microglia using sparse labeling of calyces together with broad labeling of presynaptic proteins (see section “Materials and Methods”). For the P8 endpoint, we used PV immunofluorescence to label calyces of Held. Early in development, calyces of Held are PV positive while MNTB neurons are not (Lohmann and Friauf, 1996; Caicedo et al., 1997; Felmy and Schneggenburger, 2004; Kulesza, 2014), allowing us to distinguish inputs from their target somata. Between P8 and P10, MNTB neurons also start expressing PV, making the boundary between the calyx of Held and the cell body less obvious. Therefore, in the P13 age group, we used VGluT1/2 immunofluorescence, which labeled all excitatory terminals surrounding MNTB neurons including calyces of Held. RDA-labeled calyces combined with PV or VGluT1/2 labeling allowed us to assess whether an MNTB neuron is contacted by multiple excitatory inputs or a single main input. Our RDA labeling method was sparse and extended across the VAS, allowing us to examine RDA-filled endings throughout the medio-lateral extent of the MNTB. A neuron was classified as monoinnervated if the cell was contacted just by an RDA-filled calyx of Held (Figures 1E,E’). A cell was classified as polyinnervated if presynaptic proteins on an MNTB neuron were found outside an RDA-labeled calyx (Figures 1F,F’, 6). We found that at P8 in both control and BLZ945-injected groups, the majority of neurons in the MNTB were still polyinnervated. Microglial depletion did not affect the number of polyinnervated MNTB neurons at P8 (*p* = 0.3914). As expected, in control animals the percentage of polyinnervated neurons significantly decreased with age (*p* < 0.0001). Polyinnervation also decreased with age for BLZ945-treated mice (*p* = 0.0101). However, microglia-depleted animals contained significantly more polyinnervated neurons than control animals at P13 (*p* = 0.0035; Figure 7A). To test whether BLZ945 treatment had an effect on neuronal counts, we evaluated neuronal density in the AVCN and MNTB at P13. We did not find any difference between control and BLZ945-injected animals, indicating no changes in cell number between the treatment groups in the AVCN (*p* = 0.8693; unpaired *t*-test; Figure 7B) as well as MNTB (*p* = 0.1052; unpaired *t*-test; Figure 7B). Thus, the absence of microglia prevented or slowed down the pruning of excitatory inputs from the MNTB neurons resulting in reduced monoinnervation. The observed effect was not due to the increase in neuronal numbers in the AVCN or the decrease in neuronal count in the MNTB.

We did not observe any effect of microglia depletion on the surface area (P8: *p* = 0.8840; P13: *p* = 0.8480; Figure 7C) or volume (P8: *p* = 0.9924; P13: *p* = 0.6299; Figure 7D) of calyces of Held. Calyces of Held of control animals significantly increased in both surface area (*p* = 0.0191) and volume with age (*p* = 0.0380) while there was no difference observed between calyceal sizes of microglia-depleted animals between P8 and P13 (surface area: *p* = 0.1470; volume: *p* = 0.2487; Figures 7C,D and Table 2).

### Multiple Calyces Are Found on Principal Neurons of MNTB in Untreated and DMSO-Injected Mice

**FIGURE 8:**
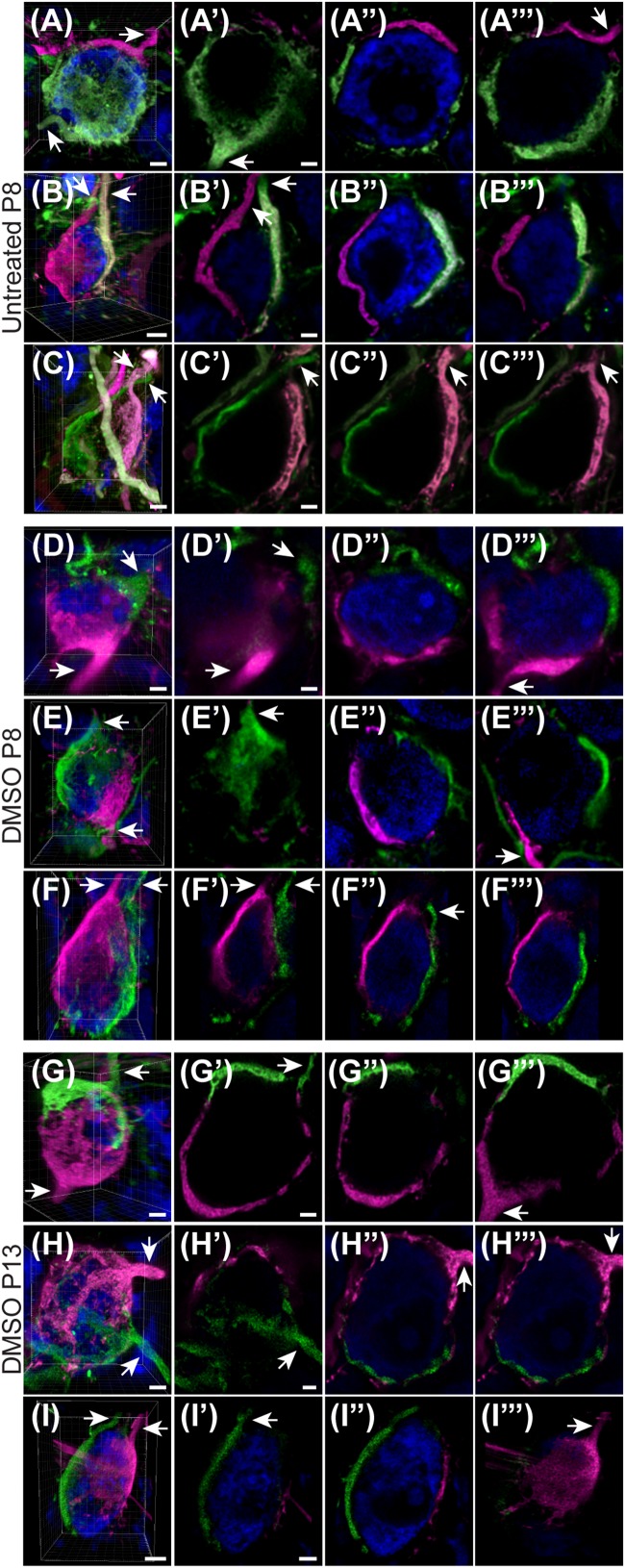
Sequential tracing with RDA and DA488 in the VAS of wild-type and DMSO-injected mice. **(A–C)** Examples of polyinnervated neurons contacted by RDA- and DA488-labeled calyces of Held in wild-type mice at P8. Arrows indicate axons. **(A’–C”’)** Individual optical sections through the neurons shown in panels **(A–C)** display polyinnervation on a single MNTB neuron. **(D–F)** 3D reconstructions of two separate calyceal inputs contacting the same neuron in the MNTB of DMSO-treated mice at P8. **(D’–F”’)** Optical sections through the neurons shown in panels **(D–F)**. **(G–I)** 3D images of reconstructed RDA- and DA488-filled calyces synapsing on a single MNTB neuron in DMSO-injected mice at P13. **(G’–I”’)** Sections through the neurons in panels **(G–I)** revealing multiple calyceal inputs. Scale bars in panels **(A,C,D)** = 3 μm. Scale bars in panels **(A’,C’)** = 2 μm and apply to panels **(A’–A”’,C’–C”’)**. Scale bar in panel **(B)** = 4 μm. Scale bar in panel **(B’)** = 2 μm and applies to panels **(B’–B”’)**. Scale bars in panels **(D,F)** = 3 μm. Scale bar in panel **(D’)** = 2 μm and applies to corresponding panels **(D’–F”’)**. Scale bars in panels **(G,H)** = 3 μm. Scale bars in panels **(G’,H’)** = 2 μm and apply to panels **(G’–H”’)**. Scale bar in panel **(I)** = 5 μm. Scale bar in panel **(I’)** = 3 μm and applies to panels **(I’–I”’)**.

Given the unexpectedly high level of polyinnervation in P8 animals in both treatment groups, we used sequential dye labeling (RDA and DA488) in untreated and control animals to verify the presence of multiple inputs. Dextrans tagged with two different fluorophores were electroporated in the VAS of untreated (*N* = 4) and DMSO-treated animals (*N* = 3) at P8. In every animal we found examples of polyinnervation (Figures 8A–F”’). Both RDA and DA488 labeled calyces were found to converge on the same neuron in the MNTB, often seen with a portion of their preterminal axons. Evaluations were only observational as injections were sparse and differed in size. We used this method to evaluate polyinnervation at P13. Although the percentage of polyinnervated neurons was only ∼10% in control animals at P13 (Figure 7A), we found examples of polyinnervation in all 3 of the double-labeled brainstems (Figures 8G–I”’).

### Microglial Association With VGluT1/2 Immunolabel

**FIGURE 9:**
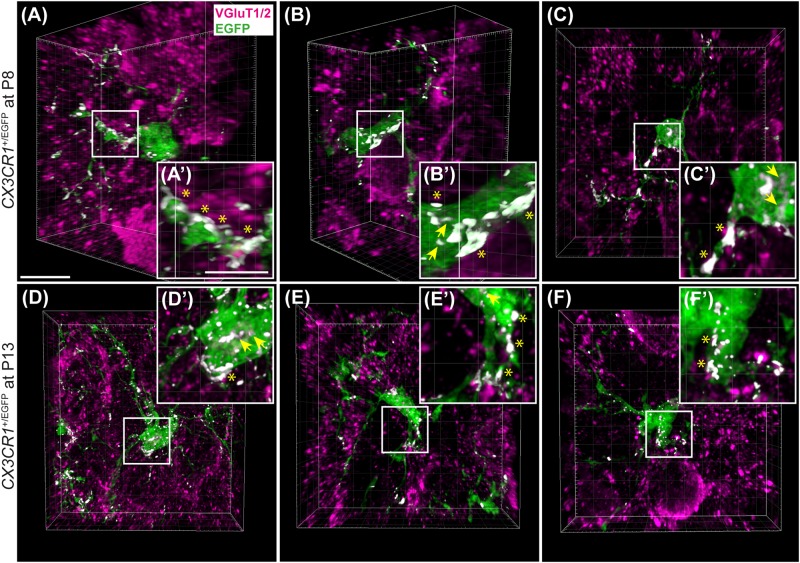
Microglial association with VGluT1/2-positive puncta. **(A–C)** 3D reconstructions of EGFP-positive microglia (green) and VGluT1/2 immunolabeling (magenta) in *CX_3_CR1*^+/EGFP^ mice at P8. White patches denote EGFP and VGluT1/2 co-localization areas. **(A’–C’)** Higher magnification images from panels **(A–C)**. Asterisks point to overlapping areas at the surface of microglial processes or cell bodies. Arrows denote internalized VGluT1/2 particles. **(D–F)** 3D confocal z-stack reconstructions of microglial cells and VGluT1/2 labeling in *CX_3_CR1*^+/EGFP^ mice at P13. **(D’–F’)** Higher magnification images of areas of co-localization between EGFP and VGluT1/2 (white). At both ages, association between EGFP and VGluT1/2 is clearly visible. Co-localization is more frequently observed at the glial surface than as internalized excitatory puncta. Scale bar in panel **(A)** = 200 μm and applies to panels **(A–F)**. Scale bar in panel **(A’)** = 5 μm and applies to panels **(A’–F’)**.

As microglia depletion was associated with impaired pruning, we next sought to determine whether presynaptic proteins are internalized in microglia during normal development. We collected tissue containing the MNTB from 3 *CX_3_CR1*^+/EGFP^ mice at P8 and P13 and immunolabeled for VGluT1/2 (Figure 9). In our analysis of five microglial cells from each animal, we found that every cell made extensive contacts with VGluT1/2-positive terminals. In addition, we consistently found small regions of VGluT1/2-labeled particles inside of the somata of EGFP-po sitive cells.

## Discussion

**FIGURE 10:**
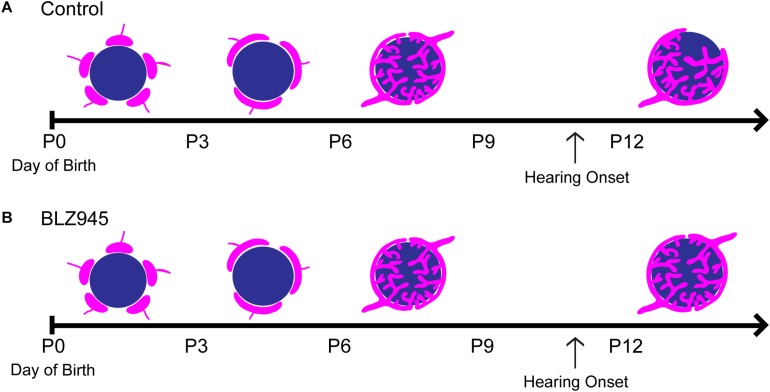
Development of the calyx of Held in control and BLZ945-injected mice. Multiple calyceal terminals converge on a single MNTB neuron early in development. **(A)** Extra inputs are pruned away until a main input emerges around the time when the ear canal opens. The dominant input grows and becomes a highly branched mature synaptic terminal. **(B)** When microglia are absent, elimination of excess calyceal inputs is reduced, resulting in greater numbers of polyinnervated MNTB neurons.

We have optimized a procedure for near complete elimination of microglia in early postnatal mice. We used this approach to test the contribution of microglia to the development of specialized neural circuits in the brainstem. We found that microglia depletion reduced the expression levels of the mature astrocyte marker GFAP, but did not affect early appearing astrocytic markers or expression levels of excitatory and inhibitory presynaptic proteins. We showed that while microglia depletion did not alter the neuronal numbers or the size of the calyx of Held, it led to impaired pruning of excess synapses terminating in the MNTB (Figure 10).

### Microglia Function in Calyx of Held Development

The function of microglia in the auditory system has been explored in the context of acoustic trauma (Baizer et al., 2015) and cochlear lesions (Campos Torres et al., 1999; Janz and Illing, 2014) but the role of microglia in developmental pruning at the large and specialized calyx of Held has not been studied. We found that at P8, both control and BLZ945-treated animals contained a rather high percentage of polyinnervated MNTB neurons, and at P13, microglial elimination resulted in significantly greater numbers of polyinnervated neurons. In the control group, the number of polyinnervated cells in the MNTB significantly decreased with age.

#### Polyinnervation of MNTB Neurons

Previous studies have described the formation of the calyx from the protocalyx into a cup-shaped structure enveloping ∼50% of the postsynaptic neuron (Morest, 1968; Satzler et al., 2002). Rapid growth of calyces during the first postnatal week was determined in the rat using light microscopy (Kandler and Friauf, 1993; Rodriguez-Contreras et al., 2008). In mice, monoinnervation was observed by P4 with the number of polyinnervated neurons decreasing by half in the second postnatal week using electron microscopy and physiological techniques (Hoffpauir et al., 2006). The presence of a single dominant input on the majority of MNTB neurons was shown by P6 and monoinnervation by P9 (Holcomb et al., 2013). In contrast, other studies demonstrated polyinnervation of MNTB neurons after the first postnatal week in rodents using physiological (Bergsman et al., 2004) and anatomical methods (Wimmer et al., 2004), although the incidence was small. Smith et al. (1998) suggested that the variation seen in the excitatory input coverage (in cat, 40–50% of neuronal surface) may be due to some MNTB cells receiving two calyces or non-calyceal excitatory inputs (Smith et al., 1998). We used PV to look for signs of polyinnervation at P8, as early in development PV is expressed in calyces (Lohmann and Friauf, 1996; Felmy and Schneggenburger, 2004; Kulesza, 2014). Thus, a PV-positive area outside the RDA labeling most likely represents an additional calyx. Moreover, in some cases we observed two calyces along with their preterminal axons converging on a single MNTB neuron. It is also possible that the smallest excitatory inputs observed outside the RDA-labeled calyx are actually non-calyceal collateral inputs from axons forming calyces on the neighboring neurons (Kuwabara et al., 1991; Hamann et al., 2003) or collaterals of axons from SBCs (Cant and Casseday, 1986). However, we occasionally observed small RDA-filled calyces along with their preterminal axons that contacted only about 25% of the neuronal surface, suggesting that small inputs could be actually small calyces rather than excitatory collaterals.

Several factors might account for the discrepancy in levels of polyinnervation using these diverse approaches. Electrophysiological techniques may underestimate the convergence of synaptic inputs due to severing of axons in brain slice preparations or shared stimulation thresholds that are hard to resolve. Additionally, electron microscopy data are challenged by the limited number of cells that can be examined. We used confocal imaging and 3D reconstruction of multiple labeled calyces from multiple animals, thus increasing the number of examples. However, we were restricted by random labeling of calyces throughout the MNTB as well as the number of calyces that fulfilled the selection criteria. It did not appear that DMSO treatment alone impaired the pruning of calyces at P8, as our sequential two-color dye electroporation revealed some examples of polyinnervation in every animal examined, with or without prior DMSO treatment. Our P13 control group showed predominant monoinnervation in the MNTB, in agreement with other studies showing that pruning largely takes place prior to this age. However, microglial depletion impaired or delayed this developmental process and resulted in significantly more neurons that were still polyinnervated by multiple calyceal synapses. Further studies are needed to determine the physiological effects of increased polyinnervation in MNTB.

#### Branching of GBC Axons

Some GBC axons can branch and give rise to multiple calyces. The branching site can be distant to (Spirou et al., 1990; Kuwabara et al., 1991; Smith et al., 1991) or near its target (Rodriguez-Contreras et al., 2006). In rodents, branching sites are rarely found outside of the MNTB (Friauf and Ostwald, 1988; Kuwabara et al., 1991; Rodriguez-Contreras et al., 2006). These studies also indicate that multiple calyces formed by the same axon contact different neurons in the MNTB. Our experiments show RDA- and DA488-labeled calyces converging on a single MNTB neuron. We do not know whether we labeled the axon coming from the same GBC and branching before the electroporation site or if the labeled axons belong to two different GBCs in the AVCN. If axon branching occurred close to its target, we would expect to occasionally encounter two RDA- or DA488-labeled calyces contacting the same neuron. However, we observed few such cases, consistent with the interpretation that labeled inputs generally arose from distinct GBCs.

### Glial Cell Interactions

Astrocyte-synapse communication plays an important role in synapse development throughout the brain. In rodent cerebral cortex, a significant period of synaptogenesis occurs at postnatal weeks two and three, the time when birth and differentiation of astrocytes is complete (Freeman, 2010; Allen and Eroglu, 2017). Astrocyte-specific transcription factors that drive heterogeneity of astrocytes are still not known (Freeman, 2010). Studies indicate that the intermediate filament GFAP is a marker for mature astrocytes (Eng et al., 1971; Pixley and de Vellis, 1984; Wofchuk and Rodnight, 1995) as well as for reactive astrocytes, as GFAP immunostaining peaks 1 week after bilateral cochlear ablation in the CN of rats (Fuentes-Santamaria et al., 2013, 2017). In the auditory brainstem, glial cell expansion coincides with neural circuit development (Brandebura et al., 2018) and astrocytic markers appear at distinct times during postnatal development. In the MNTB, ALDH1L1-positive cells are present at P0, while S100β expression is sparse and GFAP expression is completely absent. S100β expression is seen throughout the MNTB at P6, while GFAP is observed at P14 (Dinh et al., 2014; Saliu et al., 2014). Thus, it is possible that expression of specific astrocytic markers signifies distinct processes at different developmental stages.

While ALDH1L1 and S100β expression was not affected by microglia depletion, we showed a decrease in GFAP levels at P13 following BLZ945 injection. Our results differ from some previous microglial depletion studies done in adult mice where a significant increase in GFAP mRNA was found with no change in cell numbers (Elmore et al., 2014; Jin et al., 2017). Our findings also contrast with a study done in 5xfAD Alzheimer’s model mice, in which the effect of microglia depletion on GFAP expression depended on the presence of pathology and the brain region examined (Spangenberg et al., 2016). Differences may be attributed to the age of the animals as well as the specific brain region investigated (Grabert et al., 2016; Spangenberg et al., 2016; De Biase and Bonci, 2018). Previous studies demonstrated interactions between microglia and astrocytes (Walton et al., 2006; Antony et al., 2011; Reemst et al., 2016) and an astrocytic role in pruning (Chung et al., 2015; Allen and Eroglu, 2017; Bosworth and Allen, 2017). A study done in rats showed that microglia are necessary for GFAP expression in astrocytes in disease states (Liddelow et al., 2017), a characteristic that may be recapitulated during development in the MNTB. The effects of microglia depletion on GFAP expression suggest a role for microglia in astrocyte maturation. This role occurs after synaptic pruning in the MNTB is largely complete.

### Microglial Function in Synaptic Pruning

It is well established that microglia have a fundamental role in effective neural wiring of the brain through regulated apoptosis, neurogenesis (Casano and Peri, 2015; Sato, 2015; Vilalta and Brown, 2018), promotion of synaptic formation and maturation (Hoshiko et al., 2012; Miyamoto et al., 2016), and synaptic pruning in normal and pathological conditions (Paolicelli et al., 2011; Schafer and Stevens, 2015; Neniskyte and Gross, 2017). We observed that microglial processes make substantial contacts with VGluT1/2-positive puncta, and we consistently observed small regions of presynaptic proteins internalized in microglial somata. It is thus possible that microglia may be engulfing some presynaptic materials as well as identifying and tagging synapses for potential elimination by other cells. Moreover, microglia-synapse contacts may reflect trogocytosis (partial phagocytosis, or “nibbling”) of presynaptic structures (Schafer et al., 2012; Weinhard et al., 2018). This process was demonstrated in a detailed study carried out in the mouse hippocampus. The authors showed that boutons descended into the microglial cytoplasm, followed by the closure of the membrane and the subsequent trafficking of engulfed presynaptic material or axonal processes. Interestingly, the study demonstrated that the complement signaling pathway, previously shown to be involved in elimination of apoptotic cells (Iram et al., 2016) and synapses (Stevens et al., 2007; Thielens et al., 2017), is not required for trogocytosis of presynaptic elements (Weinhard et al., 2018). Whether this phenomenon generalizes to auditory system development is not clear. Further exploration of these pathways is needed to understand the mechanisms through which glial cells help to shape neural circuitry in the auditory system.

## Data Availability

The datasets generated for this study are available on request to the corresponding author.

## Ethics Statement

Animal Subjects: The animal study was reviewed and approved by University of California Irvine Institutional Animal Care and Use Committee.

## Author Contributions

GM and KC conceived and designed the study, and analyzed and interpreted the data. KG and MM contributed to analytical tools. GM, CH, and SC acquired the data. GM, CH, MM, KG, and KC drafted the manuscript. All authors took full responsibility for the integrity of the data and accuracy of the analysis.

## Conflict of Interest Statement

The authors declare that the research was conducted in the absence of any commercial or financial relationships that could be construed as a potential conflict of interest.
